# Supplementary material for: Long-Term Follow-Up of Spinal Stenosis Inpatients Treated with Integrative Korean Medicine Treatment
Source: J Clin Med. 2020 Dec 28;10(1):74. doi: 10.3390/jcm10010074 (PMC7795491; doi:10.3390/jcm10010074)
Supplement: Supplementary file 1 [file jcm-10-00074-s001.pdf]

Long-Term Follow-Up of Spinal Stenosis Inpatients Treated with Integrative Korean Medicine Treatment

Table S1. Length of hospital stay and interventions administered during the stay.

	Number of patients *N* (%)	Intervention frequencies Mean ± SD
**Length of hospital stay**		
Average	23.48 ± 17.58	
Median	21 (9, 34)	
**Intervention**		
Herbal medicine (all)	368 (97.4)	195.76 ± 156.64
Protocol decoction	341 (90.2)	74.32 ± 58.57
Protocol pills	350 (92.6)	81.17 ± 59.98
Acupuncture	377 (99.7)	40.55 ± 29.43
Electroacupuncture	372 (98.4)	37.89 ± 28.35
Pharmacopuncture	364 (96.3)	38.61 ± 31.46
Bee-venom pharmacopuncture	53 (14.0)	11.11 ± 10.11
Chuna manipulation	324 (85.7)	20.22 ± 15.08

Notes: Doctors were recommended to prescribe according to the predetermined protocol decoction; nonetheless, other herbal medicine prescriptions were allowed depending on patients′ condition and the doctor′s judgment. Length of hospital stay is presented as mean ± SD for the mean value and as 25% and 75% values for the median value.

Table S2. Changes in main outcomes at admission, discharge, and long-term follow-up.

	Admission	Discharge	Long-term follow-up
**NRS score for back pain**			
Outcome	5.73 (5.62, 5.84)	3.74 (3.62, 3.86)	3.53 (3.35, 3.70)
Change from admission		-1.99 (-2.11, -1.86)	-2.19 (-2.39, -2.00)
p-value		<.0001	<.0001
**NRS score for neck pain**			
Outcome	4.83 (4.63, 5.02)	3.80 (3.67, 3.94)	2.51 (2.30, 2.72)
Change from admission		-1.49 (-1.66, -1.33)	-2.29 (-2.57, -2.02)
p-value		<.0001	<.0001
**ODI**			
Outcome	45.62 (44.27, 46.98)	34.00 (32.63, 35.36)	28.41 (26.46, 30.36)
Change from admission		-11.76 (-13.01, -10.50)	-17.22 (-19.31, -15.13)
p-value		<.0001	<.0001
**Walking time**			
Outcome	18.76 (17.09, 20.43)		49.61 (46.16, 53.06)
Change from admission			30.41 (26.91, 33.90)
p-value			<.0001
**EQ-5D-5L**			
Outcome	0.57 (0.55, 0.59)	0.65 (0.63, 0.67)	0.80 (0.79, 0.81)
Change from admission		0.08 (0.06, 0.10)	0.23 (0.20, 0.25)
p-value		<.0001	<.0001

Notes: Analysis included nonrespondents at long-term follow-up. All values are presented as mean and 95% confidence interval. Linear mixed models were used to compute outcome changes and *p*-values. Abbreviations: NRS, numeric rating scale; ODI, Oswestry Disability Index; EQ-5D-5L, five-level EuroQol 5-dimension.

Table S3. Factors associated with improvement in the NRS score for back pain, NRS score for leg pain, and ODI.

	NRS score for back pain		NRS score for leg pain		ODI	
	**Adjusted OR (95% CI)**	***p*-value**	**Adjusted OR (95% CI)**	***p*-value**	**Adjusted OR (95% CI)**	***p*-value**
Baseline outcome	2.73 (2.13, 3.50)	<.0001	2.53 (2.03, 3.14)	<.0001	1.09 (1.06, 1.11)	<.0001
DSCA ≤70 mm^2						
	0.47 (0.25, 0.87)	0.0159	0.37 (0.17, 0.83)	0.0152	0.64 (0.35, 1.17)	0.1446
Previous lumbar spine surgery						
Yes	0.40 (0.19, 0.82)	0.0129	0.46 (0.20, 1.05)	0.0649	0.67 (0.32, 1.41)	0.2904
Neurogenic claudication (ref=no)						
Yes	0.96 (0.48, 1.92)	0.9041	0.45 (0.20, 0.97)	0.0408	1.08 (0.55, 2.15)	0.8165
Sex (ref=male)						
Female	0.33 (0.16, 0.68)	0.0024	0.97 (0.43, 2.17)	0.9339	0.49 (0.25, 0.97)	0.0397
Age	0.96 (0.93, 0.99)	0.0018	1.00 (0.97, 1.03)	0.8218	0.93 (0.90, 0.95)	<.0001
Body mass index	0.95 (0.88, 1.02)	0.1538	1.02 (0.98, 1.06)	0.4277	1.00 (0.97, 1.03)	0.9221
Smoking (ref=no)						
Yes	0.47 (0.19, 1.19)	0.1118	0.87 (0.29, 2.56)	0.7971	0.42 (0.17, 1.01)	0.0514
Drinking (ref=no)						
Yes	1.10 (0.54, 2.25)	0.7868	2.31 (0.92, 5.80)	0.0751	1.79 (0.87, 3.67)	0.1141
Walking time	1.00 (0.98, 1.01)	0.633	1.00 (0.98, 1.02)	0.994	1.01 (0.99, 1.02)	0.3688
Muscle weakness						
Abnormal	0.74 (0.31, 1.76)	0.4973	1.58 (0.51, 4.89)	0.4272	0.75 (0.31, 1.84)	0.5316
Accompanying HIVD	1.43 (0.81, 2.54)	0.2167	0.82 (0.42, 1.61)	0.5648	1.00 (0.56, 1.77)	0.999
**AUC**		0.826		0.879		0.819

Notes: Multivariate logistic regression models were used to analyze factors associated with outcome improvement. Missing values were imputed using multiple imputation. Improvement was defined as a change of more than 2 points for the NRS scores for back and leg pain and of more than 10 points for ODI. All values are presented as adjusted OR and 95% CI. Abbreviations: NRS, numeric rating scale; ODI, Oswestry Disability Index; OR, odds ratio; DSCA, dural sac cross-sectional area; BMI, body mass index; HIVD, herniated intervertebral disc; AUC, area under the ROC curve.

Multivariate logistic regression analysis was performed to identify the association between improvement in main outcomes at long-term follow-up and baseline parameters. All estimates were expressed as adjusted odds ratio (OR) and 95% CI. The following were included as covariates: values of respective outcomes at admission, dural sac cross-sectional area (DSCA), previous lumbar spine

surgery, neurogenic claudication, sex, age, body mass index, smoking, drinking, walking time without pain, motor weakness, and accompanying herniated intervertebral disc. DSCA was classified with 70 mm^2 as the reference value based on previous research [37]. The area under the curve (AUC) was presented to evaluate the goodness of fit for logistic regression models.
